# Recovery of Silver Using Adsorption Gels Prepared from Microalgal Residue Immobilized with Functional Groups Containing Sulfur or Nitrogen

**DOI:** 10.3390/ma10060636

**Published:** 2017-06-10

**Authors:** Kanjana Khunathai, Katsutoshi Inoue, Keisuke Ohto, Hidetaka Kawakita, Minoru Kurata, Kinya Atsumi, Hiroaki Fukuda, Shafiq Alam

**Affiliations:** 1 Department of Applied Chemistry, Faculty of Science and Engineering, Saga University, Honjo-machi 1, Saga 840-8502, Japan; kanjana416@yahoo.com (K.K.); ohtok@cc.saga-u.ac.jp (K.O.); kawakita@cc.saga-u.ac.jp (H.K.); 2 Research Laboratories, Denso Corparation, Komenoki 500-1, Minamiyama, Nisshin, Aichi 470-0111, Japan; MINORU_KURATA@denso.co.jp (M.K.); KINYA_ATUMI@denso.co.jp (K.A.); Hiroaki_Fukuda@denso.co.jp (H.F.); 3 Department of Chemical and Biological Engineering, College of Engineering, University of Saskatchewan, 57 Campus Drive, Saskatoon, SK S7N 5A9, Canada; Shafiq.alam@usask.ca

**Keywords:** adsorption, silver(I), microalgal residue, biofuel, chemical modification

## Abstract

Although biodiesel oil extracted from microalgae attracts much attention as one of the most promising green energies, its high production cost is a big problem, impeding its extensive use. In order to lower the production cost, the effective use of microalgal residue after extracting biofuel was investigated as a feed material of functional materials. In the present work, a new adsorbent for silver(I) was prepared by immobilizing functional groups of polyethylene-polyamine or dithiooxamide, which exhibita high affinity for soft Lewis acids such as silver(I) ions. Their adsorption behaviors for silver(I) were investigated from aqueous nitrate and acidothiourea media. The effects of the concentrations of nitrate and thiourea, as well as of sulfuric acid, were qualitatively interpreted. From the study of adsorption isotherms on these gels, they were found to exhibita higher adsorption capacity than the majority of those reported to date.

## 1. Introduction

In the coming years, biodiesel oils extracted from microalgae are expected to become a new environmentally friendly alternative energy source to fossil fuels, such as petroleum and coal, since such a diverse group of photosynthetic plants exhibits a rapid growth rate and significantly high oil content, as well as a carbon dioxide sequestration benefit [1,2,3]. However, the commercialization of the microalgae-based biofuels suffers from some problems, such as high production costs. Additionally, the treatment of large amounts of the solid microalgal residues generated in the biofuel conversion processes is another problem. If a good option is unavailable for their further utilization, such residues would become tedious wastes involving big costs for disposal, which would further deteriorate the economics for biofuel production from microalgae. From the perspective of both the environment and the economy, efforts have now been taken to convert such residual biomasses into valuable materials such as foods and fertilizers. However, there may be possibilities to convert them into more advanced functional materials, effectively making use of their unique characteristics. From such viewpoints, the authors tried to prepare novel adsorption gels for precious metals from such microalgal residue. For example, special adsorption gel exhibiting a high selectivity to gold(III) was prepared by means of a very simple method: cross-linking the residue by a condensation reaction using concentrated sulfuric acid [4]. Further, the adsorbents exhibiting a high selectivity to palladium(II) and platinum(IV) over base metals were prepared by immobilizing functional groups of polyethylene-immine (PEI) [5] and dithiooxamide (DTO) [6] onto polymer matrices of microalgal residue (MAR), taking into consideration that nitrogen and sulfur atoms belonging to soft Lewis bases exhibit a high affinity for those metal ions belonging to soft Lewis acids, according to the well-known HSAB (Hard and Soft Acids and Bases) theory [7]. These adsorption gels are abbreviated as PEI-MAR and DTO-MAR, respectively, hereafter.

As for gold, silver is now in extensive use for producing a variety of electric and electronic devices due to its high electro-conductivity. Consequently, in the recovery of metal values from spent electronics, it should be recovered together with other precious metals such as gold and palladium, as well as rare metals, such as cobalt and rare earths. As is well known, silver is barely soluble in aqueous chloride media, thus in the leaching process of precious metals from various feed materials, including scraps of spent electronics using aqua regia and hydrochloric acid blown with chlorine gas, it is leached using nitric acid in advance. Conventionally, it has been leached using an alkaline cyanide solution from primary and secondary resources, similarly to gold. However, since cyanide is strongly toxic and, consequently, technologies using cyanide solutions are not environmentally benign, non-cyanide leaching of gold and silver has been studied in recent years. One of the most promising non-cyanide leaching techniques is to use an aqueous mixture of thiourea and sulfuric acid [8,9], which is abbreviated as acidothiourea hereafter.

In the present work, the authors conducted fundamental work on the adsorption behaviors of PEI-MAR and DTO-MAR for silver(I), another typical soft Lewis acid, from nitric acid and acidothiourea solutions, by investigating the effects of the concentrations of the reactant species in the aqueous solution, on the basis of the background mentioned above.

## 2. Experimental

### 2.1. Preparation of the Adsorption Gels

**Scheme 1:**
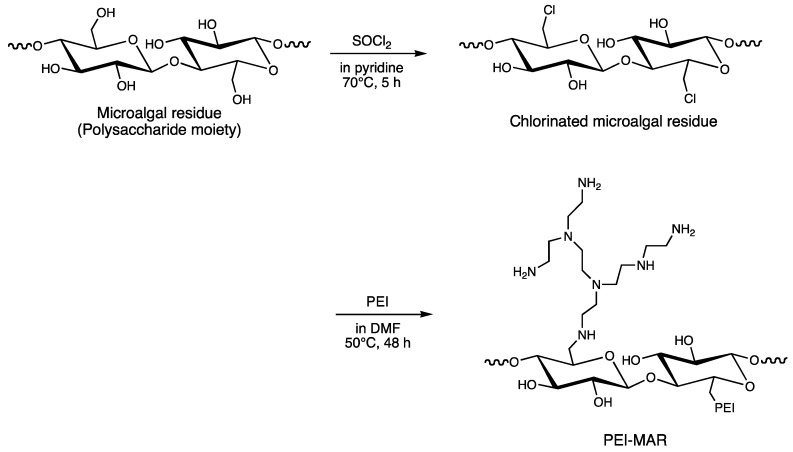
Synthetic route of PEI-MAR.

**Scheme 2:**
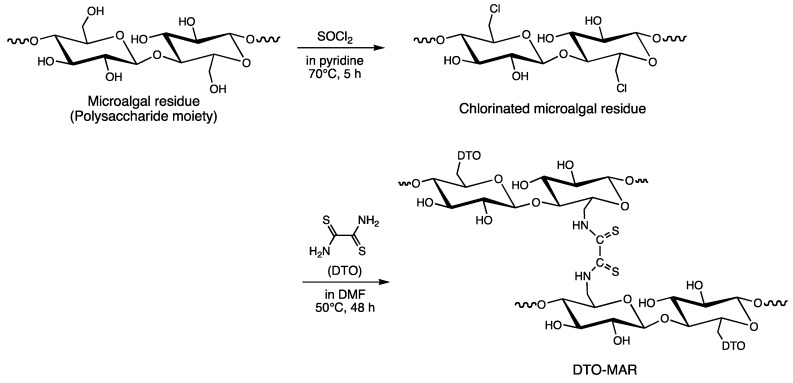
Synthetic route of DTO-MAR.

The dried sample of residual waste of *Pseudochoricystis ellipsoidea* generated in a biofuel conversion process was kindly provided by DENSO CORPORATION, Aichi, Japan. PEI-MAR and DTO-MAR were synthesized according to the synthetic routes shown in Scheme 1 and Scheme 2, respectively. The details of the synthesis as well as their characterizations are described elsewhere [5,6].

### 2.2. Preparation of Test Solutions

The stock solution of silver(I) (10 mM, M = mol·dm^−3^) was prepared by dissolving reagent grade AgNO_3_ (purchased from Wako Chemicals, Osaka, Japan) in water. Nitric acid, sulfuric acid and thiourea employed in the present work were also of reagent grade and purchased from Wako Chemicals, Osaka, Japan. In the case of adsorption tests from nitrate media, the test solutions were prepared by dissolving the stock solution into nitric acid solutions ranging from 0.1 to 5.0 M, while, in the case of those from acidothiourea solutions, the test solutions were prepared by dissolving the stock solution into mixtures consisting of varying concentrations of thiourea in 50 mM of sulfuric acid, and those consisting of varying concentrations of sulfuric acid in 50 mM thiourea, so as to maintain the Ag(I) concentration constant of 0.2 mM, except for the adsorption isotherm tests.

### 2.3. Batch Adsorption Tests

All adsorption tests were carried out batch-wise at a constant temperature of 30 °C. The gel (10 mg) was shaken together with 10 mL of the test solution for 24 h to attain an equilibrium in the adsorption tests to investigate the effects of the aqueous media. In the adsorption isotherm tests, 10 mg of the gel was shaken together with 10 mL of the test solution containing varying initial concentrations of Ag(I) (0.5–8.0 mM) in 0.1 M nitric acid, or in mixtures of 50 mM thiourea and 50 mM sulfuric acid. After shaking, the mixtures were filtered to measure the silver(I) concentration in the filtrate. The Ag(I) concentration before and after the shaking was measured using a Shimadzu ICPS-8100 ICP/AES spectrometer (Tokyo, Japan). From the concentration changes of Ag(I), adsorption percentage and the amount of adsorption of Ag(I) were calculated by Equations (1) and (2), respectively:

(1) $$\%\;\mathrm{Adsorption}=\frac{Ci-Ce}{Ci}\times 100$$

(2) $$q=\frac{Ci-Ce}{M}\times V$$

where *Ci* and *Ce* (mM) denote the initial and equilibrium concentrations of Ag(I), while *q* (mmol·g^−1^), *M* (g) and *V* (dm^3^) denote the amount of adsorbed Ag(I) on the gels, the dry weight of the gels added and the volume of the test solutions, respectively.

## 3. Results and Discussion

### 3.1. Effect of Nitric Acid Concentration

**Figure 1:**
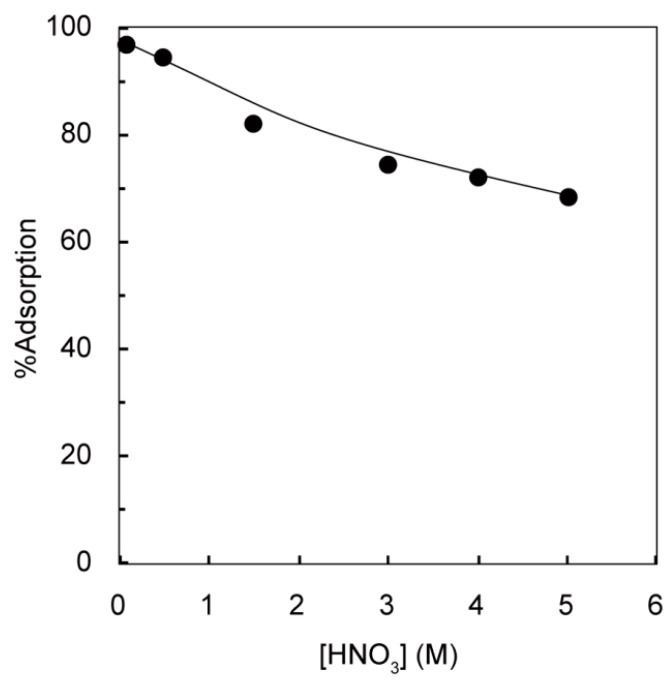
Effect of HNO_3_ concentration on the recovery of Ag(I) using the PEI-microalgae. Conditions: Ag(I) concentration = 0.2 mM, weight of adsorbent = 10 mg, volume of test solution = 10 mL, temperature = 30 °C, and shaking time = 24 h.

**Figure 2:**
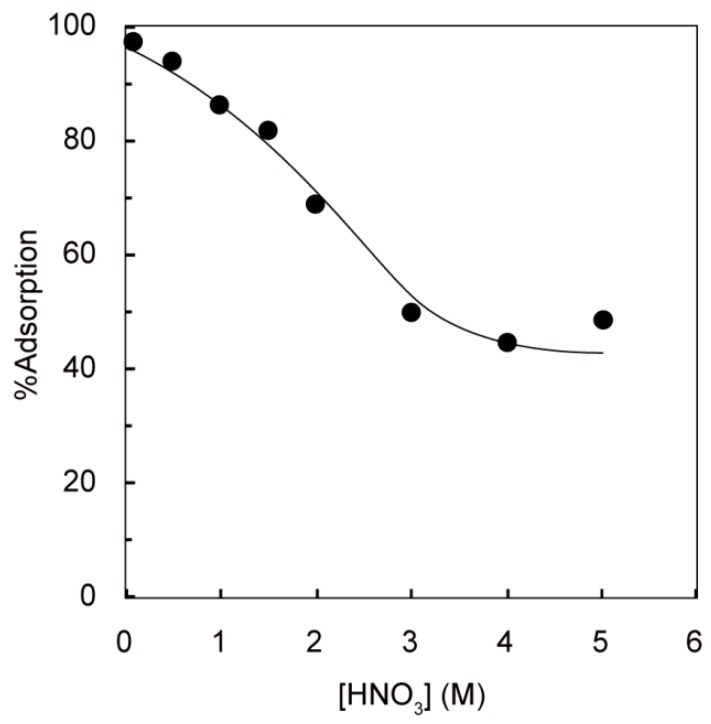
Effect of HNO_3_ concentration on the recovery of Ag(I) using the DTO-microalgae. Conditions: Ag(I) concentration = 0.2 mM, weight of adsorbent = 10 mg, volume of test solution = 10 mL, temperature = 30 °C, and shaking time = 24 h.

Figure 1 and Figure 2 show the effect of the nitric acid concentration on the adsorption percentage of Ag(I) from varying concentrations of nitric acid solution on PEI-MAR and DTO-MAR gels, respectively. As seen from these figures, the adsorption of Ag(I) decreases with increasing nitric acid concentration in both cases, which may be attributed to the formation of a nitrate complex of Ag(I) in the aqueous solution, as shown in Equation (3), that is difficult to be adsorbed when interacting with the functional groups on the gels.

Ag^+^ + NO_3_^−^ ⇄ Ag^+^NO_3_^−^ (3)

A similar effect has been observed by the authors in the solvent extraction of silver with triisobutylphosphine sulfide from nitrate media [10].

### 3.2. Effect of Thiourea Concentration on the Adsorption of Silver(I) from Acidothiourea Solution

**Figure 3:**
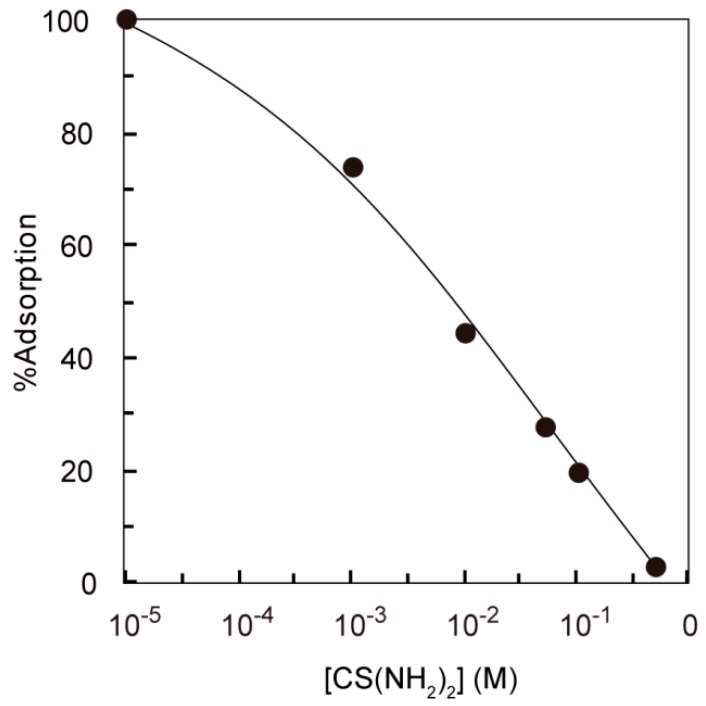
Effect of thiourea concentration on the adsorption of Ag(I) on the PEI-MAR. Conditions: Ag(I) concentration = 0.2 mM, weight of adsorbent = 10 mg, H_2_SO_4_ concentration = 0.05 M, volume of test solution = 10 mL, temperature = 30 °C, and shaking time = 24 h.

**Figure 4:**
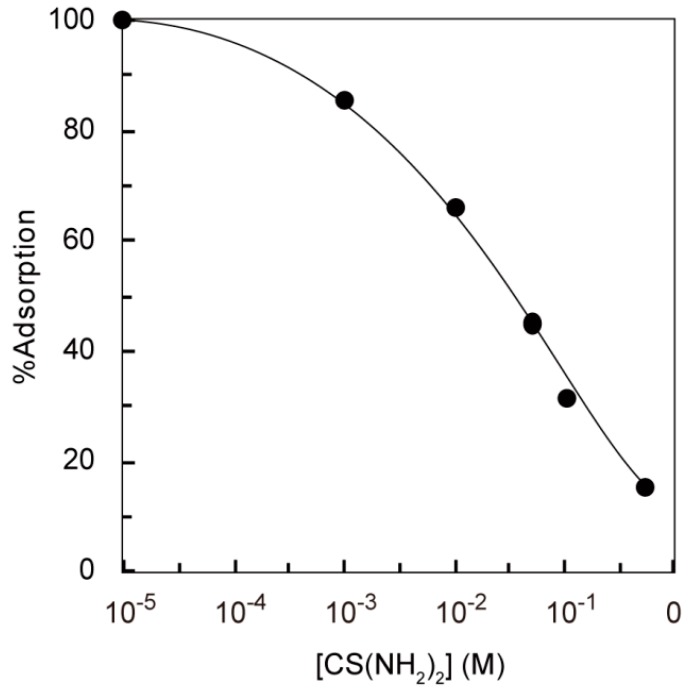
Effect of thiourea concentration on the adsorption of Ag(I) on the DTO-MAR for Ag(I). Conditions: Ag(I) concentration = 0.2 mM, weight of adsorbent = 10 mg, H_2_SO_4_ concentration = 0.05 M, volume of test solution = 10 mL, temperature = 30 °C, and shaking time = 24 h.

Figure 3 and Figure 4 show the effect of the thiourea concentration on the adsorption of silver(I) from acidothiourea solutions consisting of varying concentrations of thiourea and 50 mM sulfuric acid on PEI-MAR and DTO-MAR gels, respectively.

In both cases, the adsorptionpercentage of silver was greatly decreased with an increasing concentration of thiourea, which may be attributable to the formation of very stable complexes of Ag(I)-thiourea, which are difficult to adsorb on the gels in the aqueous solution, as shown by Equations (4)–(6):

Ag^+^ + CS(NH_2_)_2_ ⇄ AgCS(NH_2_)_2_     β_1_ (4)

Ag^+^ + 2 CS(NH_2_)_2_ ⇄ Ag[CS(NH_2_)_2_]_2_   β_2_ (5)

Ag^+^ + 3 CS(NH_2_)_2_ ⇄ Ag[CS(NH_2_)_2_]_3_   β_3_ (6)

where the stability constants of these complexes have been reported as β_1_ = 10^9.25^, β_2_ = 10^10.21^, and β_3_ = 10^13.14^ [11].

### 3.3. Effect of Sulfuric Acid Concentration on the Adsorption of Silver(I) from Acidothiourea Solution

**Figure 5:**
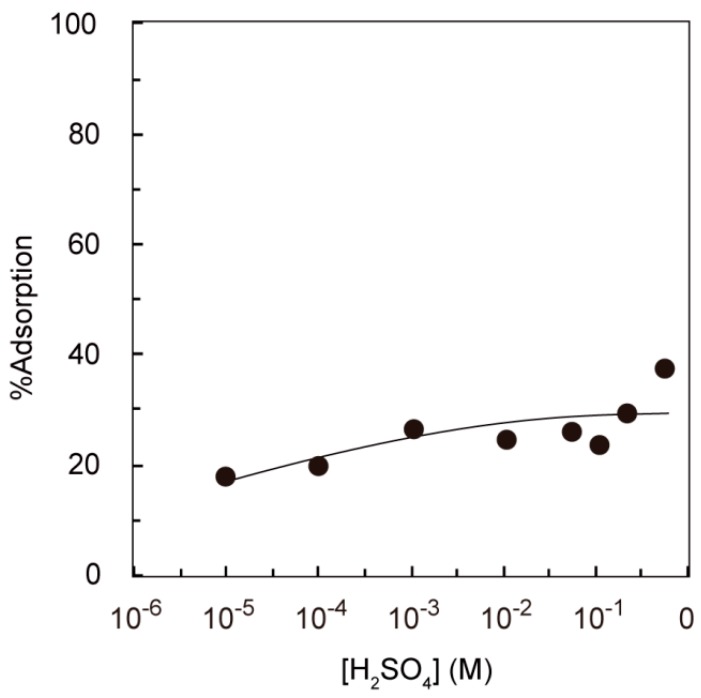
Effect of H_2_SO_4_ concentration on the removal of Ag(I) on the PEI-microalgae. Conditions: Ag(I) concentration = 0.2 mM, CS(NH_2_)_2_ concentration = 0.05 M, weight of adsorbent = 10 mg, volume of test solution = 10 mL, temperature = 30 °C, and shaking time = 24 h.

**Figure 6:**
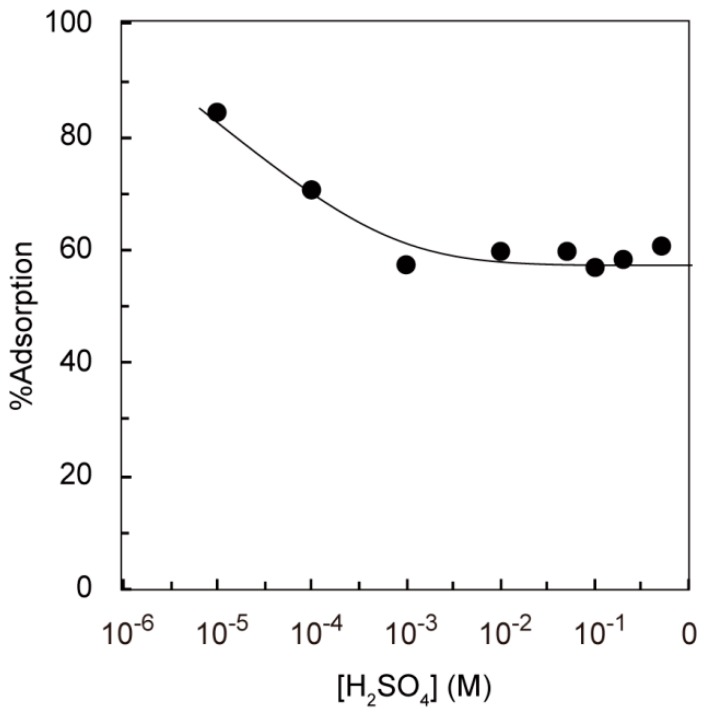
Effect of H_2_SO_4_ concentration on the recovery of Ag(I) on the DTO-microalgae. Conditions: Ag(I) concentration = 0.2 mM, CS(NH_2_)_2_ concentration = 0.05 M, weight of adsorbent = 10 mg, volume of test solution = 10 mL, temperature = 30 °C, and shaking time = 24 h.

Figure 5 and Figure 6 show the effect of the sulfuric acid concentration on the adsorption of silver(I) from acidothiourea solutions containing varying concentrations of sulfuric acid and 50 mM thiourea on PEI-MAR and DTO-MAR gels, respectively.

The adsorption of silver on PEI-MAR gelincreases slightly with an increasing sulfuric acid concentration in the low concentration range (<10^−3^ M) of sulfuric acid, while it becomes nearly constant in the higher concentration range (>10^−3^ M). On the other hand, for the adsorption on DTO-MAR gel, the adsorption decreases with an increasing concentration of sulfuric acid in the low concentration region (<10^−3^ M), but also becomes nearly constant in the higher concentration range (>10^−3^ M). That is, contrary to the effect of the thiourea concentration shown in Figure 3 and Figure 4, the concentration of sulfuric acid oppositely affects the adsorption of silver(I) in these two adsorption systems, which may be qualitatively interpreted by taking into account the protonation of thioamide groups of thiourea and DTO-MAR gel, as mentioned below.

Primary amino groups of thiourea, which are active basic functional groups, are apt to be protonated in acidic solutions, which impedes the supply of electrons to sulfur atoms of thiourea and lowers the electron density on sulfur atoms, deteriorating the coordination toward metal ions in the solution, resulting in the increase in the adsorption of silver. This is the reason forthe increase in the adsorption on PEI-MAR gel with an increasing sulfuric acid concentration.

On the other hand, for the adsorption on DTO-MAR gel, nitrogen atoms of thioamide groups in DTO functional groups are also protonated in acid solutions, which also impedes the supply of electrons to sulfur atoms of DTO functional groups and lowers the electron density on sulfur atoms, deteriorating the coordination toward metal ions. In this case, it is inferred that this deterioration is more effective than in thiourea, resulting in the decrease in adsorption with an increasing sulfuric acid concentration. This is the reason for the increase in the adsorption on PEI-MAR gel with an increasing sulfuric acid concentration.

However, because the concentration of thiourea, as well as the added amount of DTO-MAR gel, isconstant, the above-mentioned effects are limited and, consequently, the decrease or increase in the adsorption becomes nearly constant even if the sulfuric acid concentration is increased. More detailed investigations are necessary to elucidate the observed phenomena in future works.

### 3.4. Adsorption Isotherms

**Figure 7:**
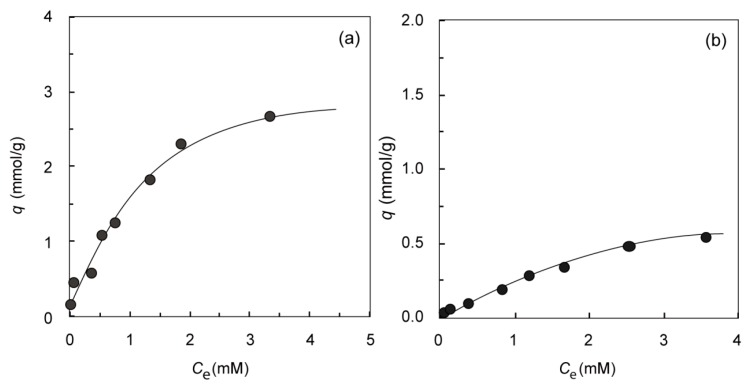
Adsorption isotherms of Ag(I) on PEI-MAR from: (**a**) 0.1 M nitric acid, and (**b**) a mixture of 50 mM sulfuric acid and 50 mM thiourea. Conditions: weight of adsorbent = 10 mg, volume of test solution = 10 mL, temperature = 30 °C, and shaking time = 4 days.

**Figure 8:**
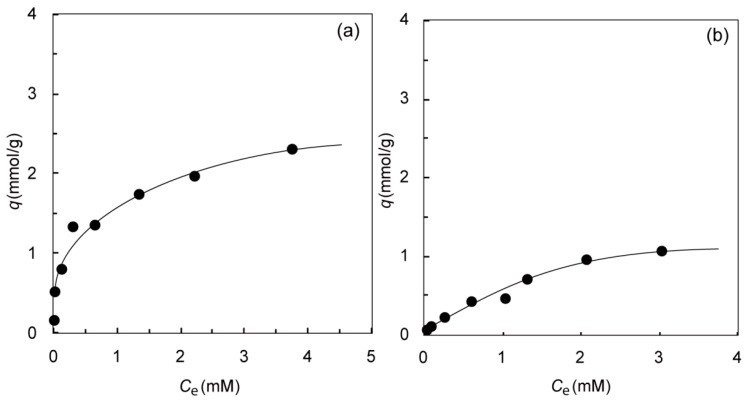
Adsorption isotherms of Ag(I) on DTO-MAR from: (**a**) 0.1 M nitric acid, and (**b**) a mixture of 50 mM sulfuric acid and 50 mM thiourea. Conditions: weight of adsorbent = 10 mg, volume of test solution = 10 mL, temperature = 30 °C, and shaking time = 4 days.

Figure 7 and Figure 8 show the adsorption isotherms of silver(I) on PEI-MAR and DTO-MAR gels, respectively. Similar Langmuir-type adsorption isotherms are observed for both gels. In both cases, the adsorption of silver(I) from the mixture of sulfuric acid and thiourea is lower than that from the nitric acid, suggesting that it is significantly impeded by the formation of the silver(I)-thiourea complex in the aqueous solution, as mentioned earlier. From the Langmuir plots for these adsorption isotherms, the maximum adsorption capacities were evaluated for each gel and each aqueous medium and compared to those of other adsorbents reported in literature, as listed in Table 1. From this table, it is evident that the DTO-MAR and PEI-MAR gels prepared in the present work exhibit higher adsorption capacities than other adsorbents, except for poly(O-phenylenediamine).

## 4. Conclusions

Novel types of adsorption gels for silver(I) were prepared by immobilizing functional groups of polyethylene-immine and dithiooxamide onto polymer matrices of the residual waste of *Pseudochoricystis ellipsoidea* after extracting biofuel to investigate the adsorption behaviors for silver(I) from nitric acid and acidothiourea. Although both nitrate and thiourea impeded the adsorption, the latter was more significant. In the adsorption from acidothiourea solutions, the effect of sulfuric acid concentration was reversed in these two gels, which was qualitatively interpreted by taking into account the protonation of nitrogen atoms of thiourea and of dithiooxamide groups. The maximum adsorption capacities of these gels were higher than for other adsorbents, except for poly(O-phenylenediamine).

## Figures and Tables

**Table 1:** Comparison of maximum adsorption capacity of various adsorbents for silver(I).

Adsorbent	Maximum Adsorption Capacity (mmol/g)	Aqueous Media	Literature
Coal	0.02	pH = 4	[12]
Expanded perlite	0.08	pH = 6.5	[13]
Clinoptilolite	0.31	pH = 4	[14]
Mesoporous silica	0.43	pH = 7	[15]
Ag(I)-imprinted chitosan	1.85	pH = 7	[16]
Poly(O-phenylenediamine)	4.94	pH = 5	[17]
DTO-MAR	2.40	0.1 M nitric acid	This work
DTO-MAR	0.98	50 mM sulfuric acid + 50 mM thiourea	This work
PEI-MAR	2.70	0.1 M nitric acid	This work
PEI-MAR	0.49	50 mM sulfuric acid + 50 mM thiourea	This work
